# Pond Sediments Reveal the Increasing Importance of Road Runoff as a Source of Metal Contamination in Industrialized Urban Environments Downwind of Pittsburgh, Pennsylvania (USA)

**DOI:** 10.1021/acsestwater.2c00240

**Published:** 2023-02-07

**Authors:** Memphis J. Hill, Daniel J. Bain, Robert J. Rossi, Mark B. Abbott

**Affiliations:** † Department of Geology and Environmental Science, University of Pittsburgh, Pittsburgh, Pennsylvania 15260, United States; ‡ PSE Healthy Energy, 1440 Broadway, Suite 750, Oakland, California 94612, United States

**Keywords:** metals, sediment, urban pond, roads, industry, vehicles, emissions, legacy

## Abstract

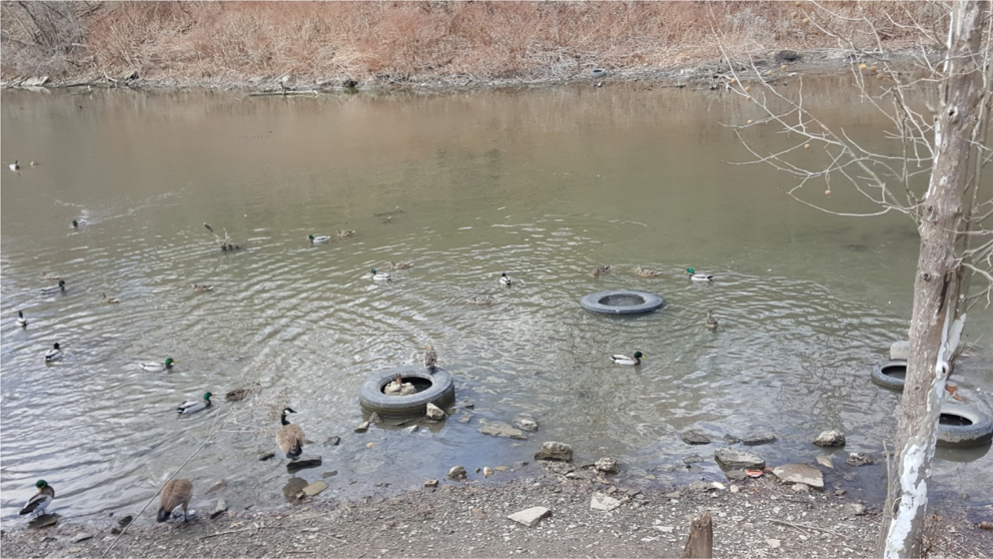

Toxic levels of trace metals from human activities accumulate in natural environments, yet these metal mixtures are rarely characterized or quantified. Metal mixtures accumulate in historically industrial urban areas and change as economies shift. Previous research has often focused on the sources and fate of a specific element, which limits our understanding of metal contaminant interactions in our environment. Here, we reconstruct the history of metal contamination in a small pond downstream of an interstate highway and downwind of fossil fuel and metallurgical industries that have been active since the middle of the nineteenth century. Metal contamination histories were reconstructed from the sediment record using metal ratio mixing analysis to attribute the relative contributions of contamination sources. Cadmium, copper, and zinc concentrations in sediments accumulated since the construction of major road arteries in the 1930s and 40s are, respectively, 3.9, 2.4, and 6.6 times more concentrated than those during industry-dominated time periods. Shifts in elemental ratios suggest these changes in metal concentrations coincide with increased contributions from road and parking lot traffic, and to a lesser extent, from airborne sources. The metal mixture analysis demonstrates that in near-road environments, contributions from modern surface water pathways can obscure historical atmospheric industrial inputs.

## Abstract 2

Pond sediments reveal road runoff as a source of industrial trace metal contamination in the cradle of the U.S. industrial revolution. The implications resonate globally for near-road systems as road networks expand.

## Introduction

Human activities have been contaminating the Pittsburgh region since European colonization. As early as 1766, personal diaries described smoke continually rising from mines in the Pittsburgh region.^1^ The Pittsburgh Coal Seam, a large bituminous coal seam underlying most of southwest Pennsylvania, crops out in Pittsburgh and has fueled local and regional glass, iron, and steel industries. Coal burning and industrial emissions were largely unregulated for almost two centuries until the implementation of local air pollution reduction policies in the 1940s.^2^ Both industrial activity and roads increase the variability and heterogeneity of metal inputs.^3−5^ These source emissions have changed over time due to regulations and advances in technology,^6−8^ yet legacy metal contamination continues to threaten water quality, human health, and ecosystem diversity.^9−11^ Understanding the legacy effects of various contamination sources requires a reconstruction of the history of metal deposition and flux over time. Metal contaminants are deposited in lake and pond sediments, forming archives that provide a window into the history of metal pollution.^12,13^

**Figure 1:**
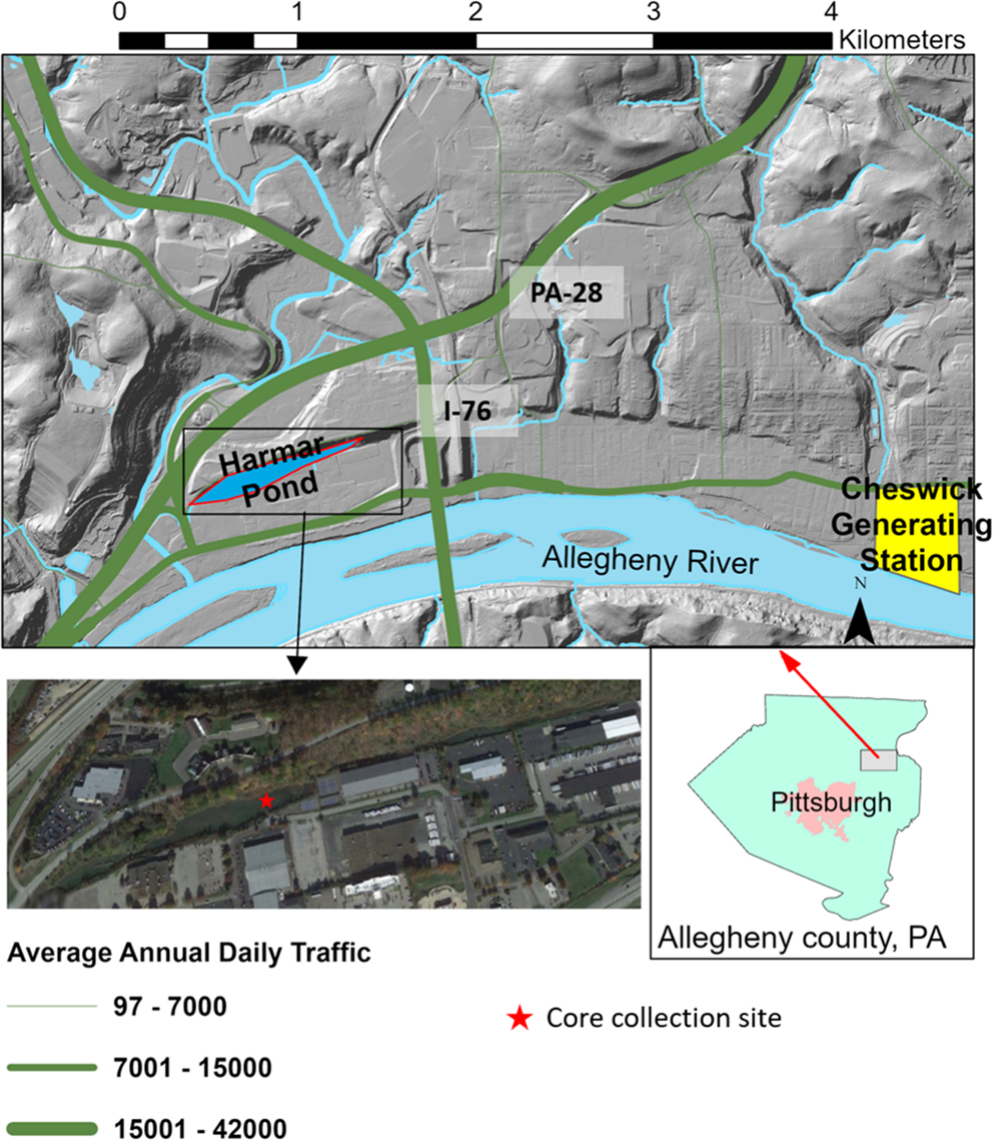
Map of the study area and the average daily traffic volume. Harmar Pond is outlined in red, not to scale, with waterways in blue, Cheswick power station in yellow, and roads in green, with line thickness corresponding to the average annual daily traffic volume. The gray box on the inset map of Allegheny County corresponds to the boundaries of the site map above. The western edge of Harmar Pond is 35 m from PA-28, and the eastern edge of the pond is 160 m from the I-76 toll plaza turn out. Seventy-five percent of the area surrounding Harmar Pond, defined by the ridge to the north, I-76 to the east, Freeport Rd to the south, and PA-28 to the west, is covered by impervious surfaces.

Examination of metal contamination histories from sediment cores reveals complex metal mixtures in lacustrine and alluvial sediments.^14−16^ Harmar Pond (Figure 1), the aquatic system explored here, has a broad variety of potential historical and contemporary metal loading sources, including, but not limited to fossil fuel combustion, vehicle emissions, metal industry emissions, and road runoff. Harmar Pond is downwind from Pittsburgh and thus received inputs from these historical industrial activities. It is also only a few kilometers upwind from the Cheswick Generating Station, a natural gas and coal-fired power plant (Figure 1), which operated from 1970 to 2022. We assembled measurements of trace metal concentrations from the literature to examine and compare the metal ratios of potential sources with the results of our measurements on Harmar sediment samples. Two ratios (Pb/Cd and Cu/Zn) were used to evaluate sediment chemistry in a mixing analysis based on urban metal mixtures^3,12−15,17−19^ and the utility of this mixing space in other environments.^20^

In addition to atmospheric metal inputs from industry, Harmar Pond receives surface water runoff from nearby roads and parking lots. Vehicle emissions release a variable mixture of metals to the environment, including both gaseous (i.e., exhaust) and solid (e.g., brake linings and tire wear) sources.^17^ The constituents of road runoff vary depending on factors such as traffic volume and patterns, rainfall intensity, seasonal changes in precipitation, road maintenance, and changes in vehicle component compositions as technology and materials evolve through time.^18^ The mixture of metals delivered to Harmar Pond is further complicated by the possibility of remobilization of legacy metals stored in catchment soils during earlier industrial activities.^19,21,22^ Here, we use a sediment core from Harmar Pond to examine sediment metal mixtures and elucidate historical and current contamination sources for this roadside aquatic system.

## Materials and Methods

A sediment core was collected from Harmar Pond, downwind from historical industrial activities in Pittsburgh and upwind from the Cheswick power plant. More importantly, Harmar Pond is flanked by two major roads (Figure 1): Pennsylvania State Route 28 (PA-28), which was constructed in 1928, and Interstate Highway 76 (I-76), which was extended to this location in 1946. Surface water inputs currently come from two main sources: parking lot runoff directed into the pond by stormwater drainage infrastructure and highway runoff from a channel upstream of the pond. This location allows the examination of the relative role of both historical industrial contamination and modern road runoff inputs. The pH of Harmar Pond water was 8.1, measured with a Hannah Instruments Piccolo Plus and averaged from three samples (7.8, 8.3, and 8.4). The prevailing winds come from the west/southwest.^23^

### Core Collection and Processing

On November 5, 2016, a 44.5 cm long intact sediment core with a diameter of 6.5 cm was retrieved from the center of Harmar Pond using a light weight percussion coring system. The corer was pushed into the sediment until refusal at the base of the sediment package (e.g., rock or thick clay). We present a single core from this pond, given its relatively small size (Figure 1). Moving from the depocenter toward the shore increases the potential for sediment mixing and moving toward inlets increases the proportion of larger grain sizes in the sampled sediment. The core was collected from the center of the pond to minimize variability in sediment size distributions and to avoid desiccation surfaces formed during dry periods. The entire core was extruded in half-centimeter increments in the field to avoid mixing during transport. The sediment was sampled using plastic instruments and Whirlpacks. The half-centimeter-thick increments were freeze-dried and stored at room temperature until geochemical analysis and ^210^Pb dating.

### Bulk Density

Bulk density was measured in extruded sediments at every fourth interval (i.e., a measurement was made every 2 cm). Dry bulk density was calculated by subtracting the weight of an average Whirlpack from the sample dry weight and dividing the difference by the sample volume, which was 16.59 cm^3^ for each of the 0.5 cm-long samples.

### Geochronology

**Figure 2:**
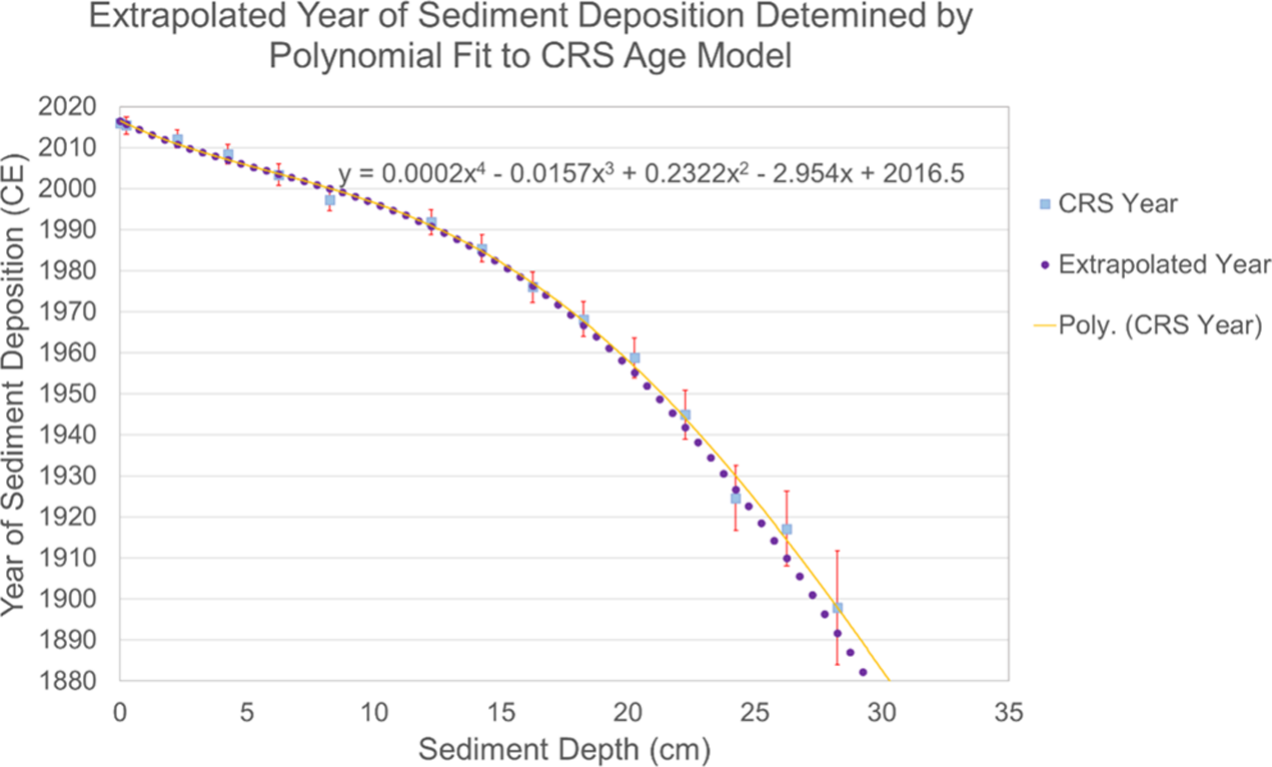
CRS age model with fit polynomial and extrapolated years of sediment deposition for each half-centimeter-thick core slice calculated using the polynomial equation. Error bars represent 68% confidence intervals.

Half-centimeter intervals of sediment from the upper 32 cm of the core were lyophilized, homogenized, sealed, equilibrated for three weeks (allowed to reach secular equilibrium), and analyzed for radioisotope (^210^Pb, ^214^Pb, and ^137^Cs) activities by direct γ counting on a high purity, broad energy germanium detector (Canberra BE-3825) at the University of Pittsburgh. Sediment ages were determined using a constant rate of supply (CRS) ^210^Pb-based dating method using the upper 29.5 cm of the core.^24^ A basal age of 1785 CE was estimated by extrapolating to the bottom of the core with a constant deposition rate. This value was then used to fit a 4th order polynomial to the CRS modeled ages (Figure 2) and resulted in a deposition date of 1882 CE for sediments at a depth of 29.5 cm. Below this depth, ^210^Pb is supported, and no other reliably datable horizons were observed, so these sediment depths are referred to as older than 1882 CE henceforth.

### Metal Analysis

Sorbed and exchangeable metals were extracted from sediment with a 10% nitric acid digest.^25^ This extraction procedure targets metals that are most likely from anthropogenic sources and are bioavailable.^25^ Metals were extracted from 100 mg (±20 mg) of sediment taken at half centimeter intervals using 6 mL of 10% (vol/vol) sub-boil distilled trace metal grade nitric acid. Extractions were shaken for 24 h and centrifuged at 4000 rpm for 15 min, and then, the supernatants were decanted into clean 15 mL polypropylene centrifuge tubes. Each acid digest was diluted 1:100 with 2% nitric acid (vol/vol), spiked with an internal standard of Be, Ge, and Tl, and analyzed for metal concentrations on an inductively coupled plasma mass spectrometer (PerkinElmer NEXion 300×) at the University of Pittsburgh. Sample duplicates and a blank were run every 10 samples to monitor reproducibility and instrument drift, respectively. Precision was within 8% for all reported elements. Metal fluxes were calculated by multiplying the concentration of a metal (μg/g) at a given depth (cm) by the bulk density (g/cm^3^) and sediment accumulation rate (cm/yr) at the same depth.

### Mixing Analysis

Metal-to-metal ratios (wt/wt) were used to infer contamination sources. We chose to focus on metal contaminants, particularly those associated with urban and urbanizing areas (i.e., Pb, Cd, Cu, and Zn).^12,17,18,26−28^ Moreover, this suite of metals has been previously used to clarify mixes of sediment sources to lakes.^20^ Here, we apply these metals to a distinct question, analysis of contaminant source contributions to sediment chemistry. We define the mixing space using the wt/wt ratios of copper to zinc (Cu/Zn) and lead to cadmium (Pb/Cd) based on previous applications.^20^ We use these ratios to avoid the accounting required to track dilution/enrichment in sediment chemistry during mixing. The analysis presented here is the first to apply the Cu/Zn versus Pb/Cd mixing space to the evaluation of contaminant sources in lake sediments. Cu/Zn and Pb/Cd ratios were plotted against each other for each Harmar sediment sample. Cu/Zn and Pb/Cd of road runoff, road dust, coal, coke, coal fly ash, and zinc smelter emissions endmembers were plotted in the same mixing space (Figure 5). Endmember source chemistries were based on values taken from a synthesis of the peer-reviewed literature (Tables 1, and S2). These endmember ratio clusters were then compared with the Cu/Zn and Pb/Cd of the Harmar sediments for 89 depth intervals to evaluate the relative contributions of each source.

**Table 1:** Endmember Region Maximum and Minimum Pb/Cd and Cu/Zn Ratios

	road runoff^51−55^			road dust^56−61^			fly ash/flue gas^62−64^		
	locations: USA			locations: USA, Nepal, China, and Canada			locations: USA and Italy		
	*n* = 831			*n* = 905			*n* = 16		
	average	min	max	average	min	max	average	min	max
Pb/Cd	37.2	2.0	82.0	175.5	46.0	420.0	89.3	0.5	235.0
Cu/Zn	0.1	0.3	0.6	0.3	0.1	0.7	0.9	0.5	1.4

	Pittsburgh Coal^64^			lower freeport coal^64^			coke emissions^65,66^		
	locations: USA			locations: USA			locations: USA		
	*n* = 6			*n* = 4			*n* = 25		
	average	min	max	average	min	max	average	min	max
Pb/Cd	62.7	42.9	84.9	106.5	80.4	132.6	19.7	9.1	27.3
Cu/Zn	0.5	0.3	0.8	0.5	0.2	0.7	0.2	0.2	0.3

	Monaca emissions^32^			Palmerton emissions^34^			Donora emissions^33^		
	locations: USA			locations: USA			locations: USA		
	*n* = 11			*n* = 10			*n* = 25		
	average	min	max	average	min	max	average	min	max
Pb/Cd	21.2	3.4	40.9	17.83	N/A	N/A	1.31	0.18	4.62
Cu/Zn	0.00073	0.000026	0.0074	0.0517	N/A	N/A	N/A	N/A	N/A

## Results and Discussion

### Geochronology

Radiometric dating and archival research suggest the sediment core captures deposition from ∼1882 to 2016 CE (Figure 2). The basal age of the core is 1785 CE but is only an estimate because the half-life of ^210^Pb (∼23 yr) limits the reliability of the age model to ∼120 years.^25^ Fortunately, the major changes in trace metal stratigraphy occur within the last 100 years (e.g., Pb, Cd, Zn, and Cu concentrations increase around 1930 CE). The ^210^Pb profile shows a steady exponential decrease with depth as sediment compaction takes place and indicates consistent and uninterrupted sediment deposition with minimal, if any, disturbance (Table S1). Sediment properties (e.g., bulk density, Figure S1) change gradationally across unit boundaries, consistent with continuous deposition.

### Metal Concentrations and Fluxes

**Figure 3:**
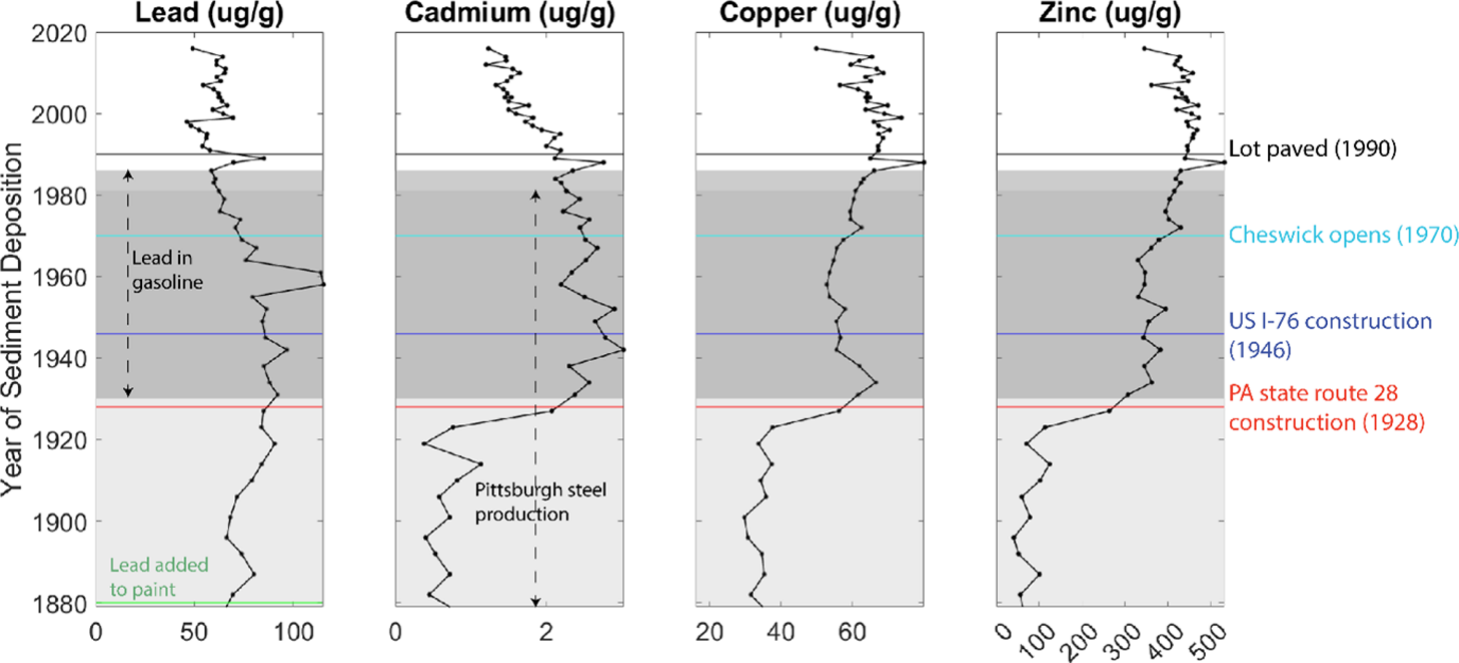
Lead, cadmium, copper, and zinc concentrations in the Harmar Pond sediment core and lines indicating relevant historical changes. The black line, cyan line, blue line, and red line indicate the paving of the adjacent lot to Harmar Pond (black), the beginning of operations at Cheswick Generating Station (cyan), construction of the western extension of I-76 (blue), and the construction of PA-28 (red). The green line on the lead plot indicates when Pb became a common addition to paints.^29^ The dark gray shaded region on the plot indicates when lead was used as a gasoline additive from 1930 to 1986 CE.^30^ The lighter gray shaded region of each plot outlines Pittsburgh steel production and bust, 1875 to 1982 CE.^31^

Sediment zinc, copper, and cadmium concentrations all increase after road construction (Figure 3). Copper and zinc concentrations continued to increase after 1946 CE and then leveled out in the 1990s. Lead concentrations gradually increase beginning in the late 1800s, reach a maximum around 1958 CE, and then gradually decrease. Zinc, Cu, and Cd concentrations are all elevated in the most recent 60 years of the sediment record relative to concentrations prior to the 1920s. Cadmium increases in a similar manner to Cu and Zn but gradually begins to decrease after reaching a maximum concentration in ∼1941 CE. There is then a sediment interval with elevated concentrations of all four metals deposited around 1989 CE.

PA-28 was constructed in 1928 CE, roughly during the transition in Harmar Pond from industry-dominated metal contamination to road-dominated metal contamination. The increase in cadmium, copper, and zinc concentrations in Harmar sediments after this transition in the contamination source was calculated by dividing the mean concentrations of each metal in sediments deposited after 1928 CE by the mean concentration of sediments deposited before 1928 CE. Harmar sediments deposited during the road-dominated period contain an average of 3.9 times more Cd, 2.4 times more Cu, and 6.6 times more Zn.

**Figure 4:**
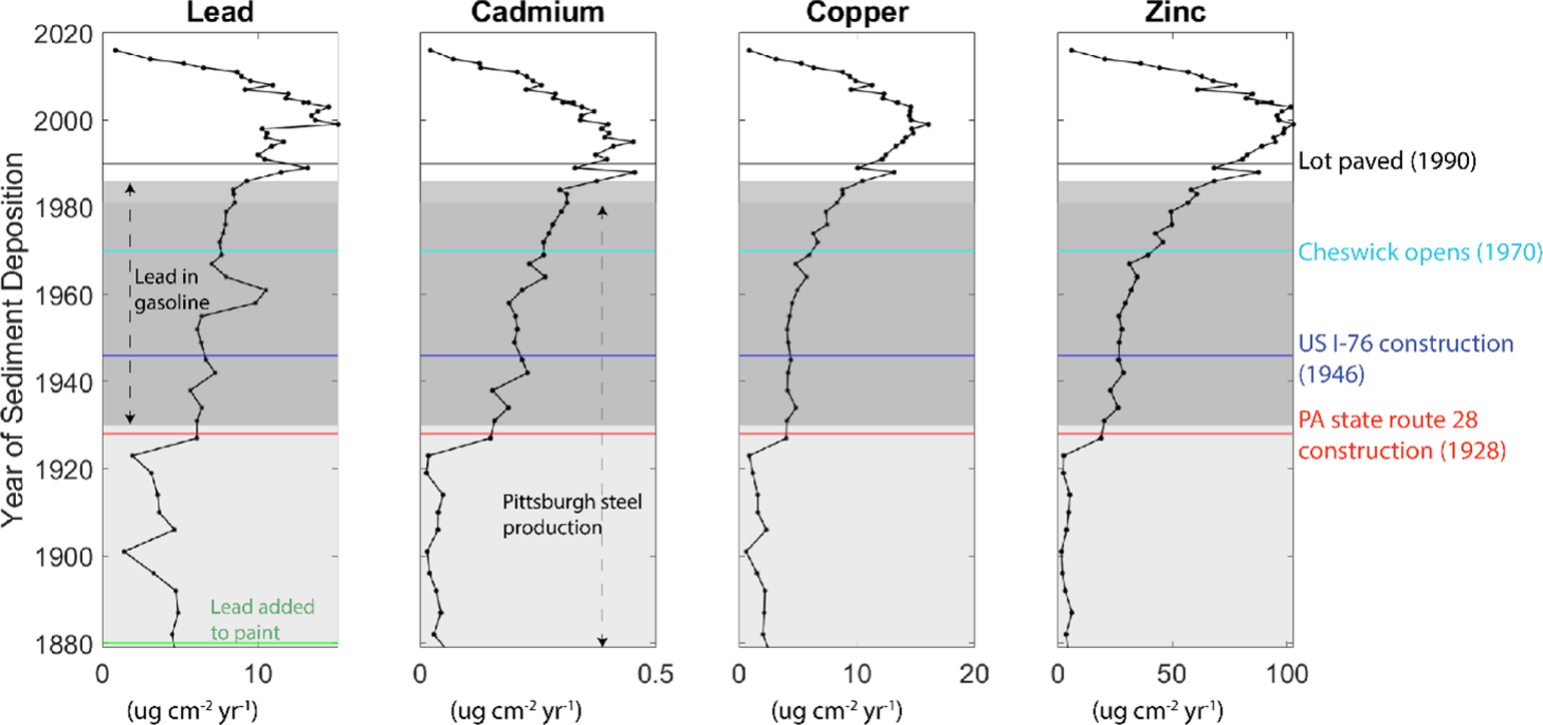
Lead, cadmium, copper, and zinc fluxes in the Harmar Pond sediment core and lines indicating relevant historical changes. The colored lines indicate the paving of the adjacent lot to Harmar Pond (black), the beginning of operations at Cheswick Generating Station (cyan), construction of the western extension of I-76 (blue), and the construction of PA-28 (red). The green line on the lead plot indicates when lead became a common addition to paints.^29^ The dark gray shaded region on the plot indicates when lead was used as a gasoline additive from 1930 to 1986 CE.^30^ The lighter gray shaded region of each plot outlines Pittsburgh steel production era from 1875 to 1982 CE.^31^

Although only lead, cadmium, copper, and zinc are presented here, several other vehicle-related metals, including nickel, cobalt, and chromium, have similar patterns to those observed in copper and zinc (Figure S2). Lead, Cd, Cu, and Zn fluxes increased in the late 1920s, and again, in the 1960s. Fluxes peaked around the year 2000 CE (Figure 4).

### Endmember Mixing Analysis

All Harmar sediments deposited after ∼1927 CE have low variability in Cu/Zn ratios, less than 0.22 and greater than 0.14, and low variability in Pb/Cd ratios, less than 53 and greater than 24. Older Harmar sediment metal ratios are generally higher and more variable. The two metal ratios converge to a narrow, low value range around the time that Pennsylvania state road 28 (PA-28) was constructed in 1928 CE.^35^ Literature ratios were calculated from mean metal values whenever possible. Details on these sources are presented in Table S2.

## Discussion

### Timing and Sources of Metal Contaminants to Harmar Pond

Metal concentrations in Harmar Pond sediments suggest that the dominant anthropogenic sources shifted from industrial to vehicle emissions after major road construction in the mid-20th century. Endmember chemistries for a variety of potential metal sources were synthesized: coke emissions,^36^ lead paint, road dust, road surface water runoff, coal from local mining operations, fly ash emissions from coal-fired power plants, and regional zinc smelter emissions. Metal concentrations of the dated Harmar core are first compared to a timeline of local land-use changes,^31,35^ as shown in Figure 3, to reconstruct the metal contamination history of the pond. This reconstruction was then refined by comparing the metal ratios of potential metal contamination sources to the metal ratios observed in Harmar sediments. Changes in metal profiles are also dependent on processes external to the sources; for example, metal inputs can be diluted during periods of increased erosion and thus appear diminished. Metal ratios are used to avoid the influence of dilution on metal concentrations.

### Timing of Regional Industrial Emissions Inputs to Harmar Pond

**Figure 5:**
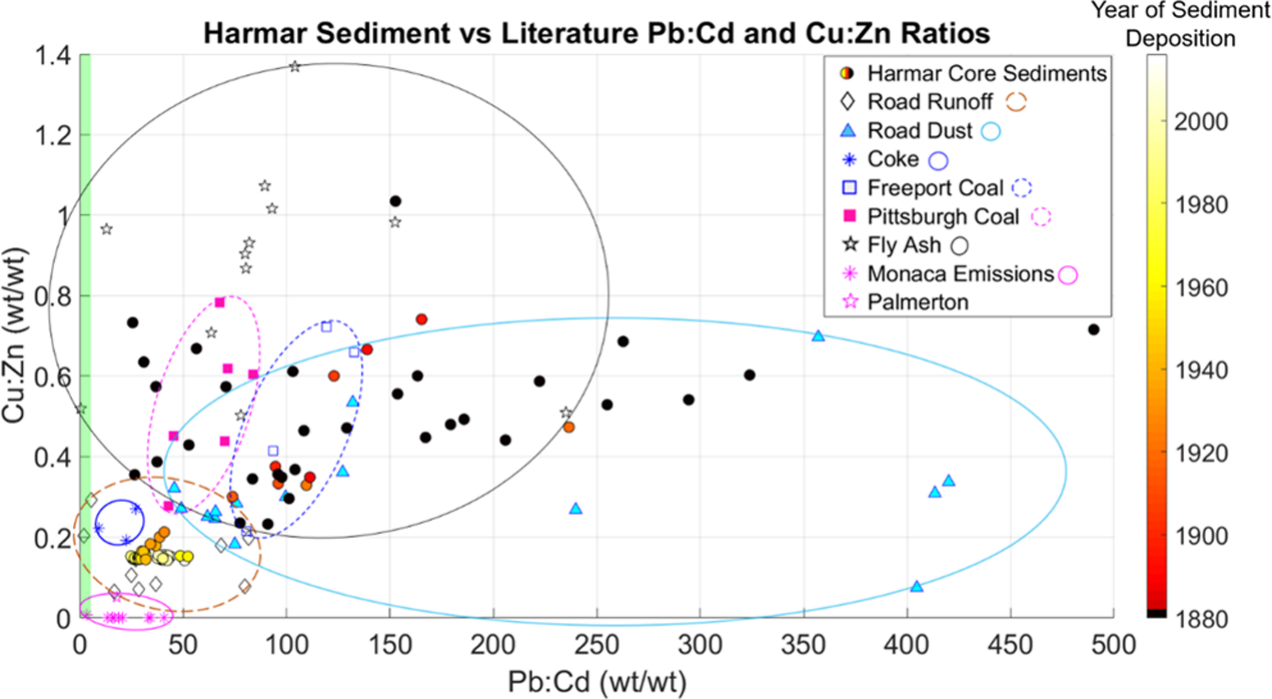
Lead to cadmium (Pb/Cd) ratios plotted against copper to zinc (Cu/Zn) ratios in Harmar Pond sediments and ranges of potential endmembers. Ratios are wt/wt. Harmar sediment data are circles colored by age according to the color bar on the right; all sediments deposited on or before 1882 CE are in black. Literature-derived data are shown as diamonds for road runoff, light blue triangles for road dust, blue asterisks for coke, pink squares for Pittsburgh coal, blue outlined squares for Freeport coal, stars for coal fly ash, pink asterisks for Monaca emissions, a pink star for Palmerton emissions, and a green line for Donora emissions. Seven regions encompassing the literature-derived data points from each source are encompassed by a dashed orange oval for road runoff, solid light blue oval for road dust, dotted pink oval for Pittsburgh coal, dotted blue oval for Freeport coal, solid blue oval for coke, solid black oval for fly ash, and solid pink oval for Monaca and Palmerton atmospheric emissions. Metal contents for the Monaca Emissions endmember collated from EPA’s yearly Toxics Release Inventory report,^32^ which only reported copper in 1998, 1999, 2003, 2004, 2005, 2006, 2007, 2008, 2009, and 2010. The Monaca emissions endmember consists of data from every year that copper was reported in the EPA TRI reports for a total of *n* = 11. The literature on Donora emissions did not report copper concentrations, so the green line represents the range of Pb/Cd ratios (0.18 to 4.6) calculated from Schrenk et al. (1949)^33^ for a total of *n* = 25. The Palmerton^34^ data point is calculated from metal emissions averages, where *n* = 10, and the zinc smelter endmember oval is drawn to include both the Palmerton and Monaca emissions ratios.

Prior to ∼1928 CE, metal concentrations in the sediments varied and may correspond with regional dynamics in industrial production interacting with weather patterns, specifically the predominant winds from the west/southwest. Some Harmar sediments deposited in the late 19th and early 20th centuries have metal ratios within the coal and coal fly ash regions of the mixing space (Figure 5). The Pittsburgh steel production was a dominant industry from the late 19^th^ century until the 1980s, when 75% of Pittsburgh’s steel mills closed.^37^ The rise in Pittsburgh steel production coincides with an increase in lead but not in Cd, Cu, or Zn in Harmar Pond. If steel production emissions dominated metal inputs to Harmar Pond, sediment metal content should also decrease as the steel industry waned. Such a shift is apparent in records from lakes that are closer to industrial facilities,^14^ but not in the Harmar record (Figure 3). Specifically, the Harmar sediment record reflects continuous metal loadings after the steel industry decline, suggesting that other contamination sources continued environmental contamination legacies. Although Cd, Cu, and Zn concentrations may be elevated relative to background levels in soils exposed to steel industry emissions, recent studies have shown that these metals are present in concentrations too low to explain those found in Harmar sediments.^26^

Coal fly ash chemistry is used as a general proxy for regional and local industrial emissions, as most industries were likely coal-powered during this historical period (1830–1915 CE). Historical industries close to Harmar Pond included paint and glass manufacturing in the late 1800s (Figure S3). Unfortunately, the metal contents of historical emissions from the paint and glass industries are poorly documented. Sanitary engineers during that period had just started to focus on the human health impacts of industrial waste discharges to surface waters.^38^ They recognized the myriad of inputs, yet the specific chemical fluxes were not measured.^39^ Lead content of paint chips has been measured;^40^ however, paints used purified lead, elevating lead relative to cadmium well above those ratios observed in Harmar sediments, and the ratios of copper to zinc in lead paint are much lower than those observed in the Harmar record (Table S2).

Two other potential metal inputs, AMD and coal from local mining, are not likely contributors to Harmar Pond. Although the Harmar Coal Mine operated close to Harmar Pond from 1915 to 1980 CE, it is not hydrologically connected to the pond. This period of mine operations does overlap with elevated lead concentrations in Harmar sediments (Figure 3), but the pond is topographically higher (∼5 m) than the mine-impacted tributary (Deer Creek). A ∼33 m high ridge divides the pond from Deer Creek making subsurface connectivity unlikely and minimizing the likelihood of coal waste or acidic mine drainage inputs from the Harmar Coal Mine to Harmar Pond. In addition, the color of Harmar Pond sediment (brown) and pH of the water (8.1) are not consistent with AMD inputs (usually a distinct rusty orange color and low pH). Further, barium (Ba) concentrations are relatively consistent in Harmar sediments (Figure S4). If AMD inputs had occurred, Ba concentrations would be the highest in sediments deposited following mining operations due to the deposition of barium sulfate precipitates.^14^ The observed metal ratios in Harmar sediments post-1930 CE do not extend into the coal regions of the mixing space. Any coal dust atmospherically deposited into Harmar Pond since 1930 CE is hard to detect. The coke region of the mixing space is close to the post-1930 CE Harmar sediments but does not overlap. Lead paint was excluded from the mixing space because the median Pb/Cd value was 1305.5, which would skew the x-axis.

Finally, two zinc smelters operated approximately 40 km from Harmar Pond: Donora Zinc Works (1915 to 1957)^41,42^ to the south and Monaca Smelter (1931 to 2014)^43^ to the northwest. Although the opening of the Monaca Smelter coincides with the observed increases in metals in core sediments, the closing of neither facility matches well with decreases in observed metal concentrations. Though the similarities in timing could suggest pond sediment chemistry changes due to smelting activity, it is difficult to imagine a system where only the Monaca Zn activity influences core chemistry while closer and earlier facilities, e.g., the large coking facilities near Pittsburgh (Clairton, LTV, and Shenango), are not necessarily apparent in the sediment chemistry record.

### Local Road Influence

The most prominent shifts observed in Harmar Pond sediment Cd, Cu, and Zn concentrations occur between 1927 and 1942 CE. The rise in Cd, Cu, and Zn concentrations occurs in sediments dated to ∼1927 CE by the extrapolated years based on the polynomial fit to the CRS age model. At this point in the past, the age model has a 1-σ, or 68% confidence interval, of ±7 years. Major roads, PA-28 and US I-76, were constructed less than 200 m from the pond (Figure 1), in 1928 and 1946 CE, respectively.^35,44^ Also, Gulf Oil opened a research facility just north of the pond in 1935 CE,^45^ which likely increased traffic on PA-28. Construction of the western extension of US I-76 from Irwin to Pittsburgh began in the 1940s and opened to the public in 1951 CE.^44^ The fluxes of Pb, Cd, Cu, and Zn all increased first in the 1920s and then again in the 1950s and 60s (Figure 4). The increases in copper and zinc flux continued until the 2000s (Figure 4), consistent with the increase in traffic on US I-76 in the mid to late 1900s.^44^ Atmospheric deposition from vehicle emissions and surface runoff from these roads seem to have provided new sources of metals for the pond.

The metal content of vehicle exhaust and nonexhaust sources has changed over time. These changes in metal content seem to be reflected in the Harmar sediment record (Figure 3). Unlike Cu and Zn, cadmium decreased in Harmar sediments throughout the 1990s and into the 2000s. Historically, Cd was once a common polymerization catalyst in tires,^46^ but over time, Cd was replaced by Zn.^47^ Zinc and Cu concentrations spike in the late 1980s and remain elevated throughout the upper portions of the core (Figure 3). These metal concentration patterns are consistent with non-exhaust vehicle emissions being the dominant source of Cu and Zn to Harmar Pond. Brake pads and tires contain copper and zinc, respectively,^17^ and erode when drivers slow down. Drivers frequently engage their brakes in parking lots. The Gulf Oil Research facility parking lot is less than 200 m north of Harmar Pond, and since 1990 CE, the southern bank of the pond has been lined by a shopping plaza parking lot. In addition, directly west of the pond is the US I-76 exit 11 toll plaza,^35^ where drivers are required to slow down from 60 to 0 mph, causing additional wear on brake pads and tires.

Lead concentrations in the Harmar sediment record reflect historical changes in lead emissions from automobiles. Lead was used as an additive for petroleum nationwide from the 1960s until 1986 CE.^30^ The highest lead concentrations in Harmar sediments occur during this period, between 1965 and 1973 CE. Lead concentrations in Harmar sediments began to decrease in the late 1970s, coinciding with the introduction of the catalytic converter. Automobile industries began installing catalytic converters on most vehicle models in 1975 CE to comply with federal regulations to reduce the atmospheric emission of metals from vehicle exhaust.^48^ Federal policy banned Pb additives with the Air Lead Reduction Act of 1984.^49^ This act called for Pb additives to be phased out starting in 1986, followed by a complete ban on the sale of gasoline with Pb additives for highway use by 1995.^50^ Lead concentrations in the Harmar record decreased in the 1980s and 1990s mirroring this history of lead regulation. Interestingly, lead flux does not follow the same regulation-driven timeline as lead concentrations. All four metal fluxes steadily increased from the late 1960s until around 2000. This is perhaps due to increased erosion of near-road Pb-rich sources and sustained increased fluxes.

### Metal Ratio Mixing Space Clarifies Inputs

The sediment metal chemistries observed in Harmar Pond were compared to contamination sources in a metal ratio mixing space with endmembers defined based on literature values. Plotting metal ratios in this Pb/Cd versus Cu/Zn mixing space reveals dominant contamination sources and how the mix of inputs changed over time. Most notably, Harmar Pond sediment chemistries deposited after 1927 CE shift to a chemistry similar to literature values for the road runoff endmember.

Harmar sediments deposited before 1927 CE have a wide range of Pb/Cd and Cu/Zn values varying across chemistries similar to fly ash and coal (Figure 5). In contrast, after 1927 CE, Harmar sediment chemistries coalesce into remarkably narrow ranges. These post-1927 CE sediments cluster in a region between 0.14 and 0.22 Cu/Zn and between 24 and 55 Pb/Cd, a range that falls completely within the literature values of observed road runoff chemistry (Table 1). The opening of PA-28 in 1928 CE and the Gulf Research Lab parking area in 1935 CE increased vehicular traffic near Harmar Pond. When construction of I-76 began in 1946 CE, Harmar sediment Cu/Zn and Pb/Cd ratios clustered even more closely within the road runoff region of the mixing space. Together, these observations strongly indicate that road contributions have been the dominant source of contamination since the 1930s. The Harmar sediment record supports conclusions made by other researchers in urban watersheds; particles in runoff from impervious surfaces (e.g., roads) are a major source of contaminants in aqueous systems.^67^

### Zinc Smelter Endmember Regions

The distance between Harmar Pond and the zinc smelters in southwest Pennsylvania is approximately 40 km, likely beyond the zone of appreciable chemical inputs from Zn smelter emissions.^68^ The Cu/Zn ratios from Palmerton and Monaca emissions are low due to the high concentration of Zn in these emissions (Figure 5). The smelter endmember region does not overlap with any of the Harmar Pond sediment chemistries that we attribute to a road influence (Figure 5). Therefore, despite noted similarities in timing, it is unlikely these observations are due to Zn smelting inputs.

### Local Coal-Fired Power Plant’s Influence on Core Chemistry

One surprising aspect of the observed sediment chemistry is that sediment metals are not clearly sensitive to operations at the Cheswick Power Station, despite its proximity to Harmar Pond (Figure 1). Cheswick, a natural gas and coal-fired power plant, started operations in 1970 CE and began implementing NOx emissions controls in 1993 CE. Trace metal concentrations do not change appreciably during these periods. The rise in metal fluxes increases even more so after Cheswick opened (Figure 4); however, the mixing analysis of Harmar sediment chemistry after 1970 CE does not seem to indicate substantial contributions of chemistries similar to coal fly ash (Figure 5).

Road runoff seems to be the major source of metal contamination in Harmar Pond sediments since ∼1930 CE. Metals from vehicle exhaust and vehicle part erosion accumulate on the surface of roads, increasing the concentration of soluble and exchangeable metals in pavement sediment.^67^ These metal-enriched pavement sediments and associated water solutions are then delivered to surface waters during storm events. Research on other pollutants in lakes, such as polycyclic aromatic hydrocarbons, has also shown that roads and vehicles are dominant sources of contamination.^69^ Globally, ecosystems with a wide variety of sensitivities are likely to be impacted by road runoff contamination.

## Implications and Limitations

Patterns in Harmar sediment metal ratios indicate that road runoff strongly influences chemical changes recorded in aquatic sediments located adjacent to major road systems. Other regional lacustrine records situated further from roads indicate the majority of contamination continues to be from industrial activities throughout their entire records. This is not surprising as industrial contamination is spread through the atmosphere across wide areas while Harmar Pond contamination is concentrated, through direct surface runoff from roadways. The sediment record from Harmar Pond reflects the efficacy of regulations, such as the ban on leaded gasoline in the 1980s; however, metals from road runoff continue to accumulate in this pond. The mixing analysis used here demonstrates a shift in the metal contamination source, and comparisons with potential contaminant endmembers revealed that road runoff inputs dominate the pond since road construction in the 1930s and 40s. This method, applied to other near-road aquatic ecosystems, can clarify road contamination inputs. This type of work can allow for predictions of how road runoff metal mixtures will continue to evolve with changing economies and technological advances.

Our work suggests that aquatic systems connected to roads or paved surfaces like parking lots will be subjected to similar metal loadings as those observed in Harmar Pond. The convergence of sediment metal chemistry toward road runoff source mixtures has widespread potential implications for biodiversity and ecosystem function in near-road aquatic environments. For example, metal contamination in sediments may select for metal-tolerant microbes and shift the composition of environmental microbiomes.^70^ Identifying the signature metal mixtures of runoff contaminated sediments is just the first step toward understanding the long-term impacts of these metal mixtures on aquatic ecosystems. Our findings in Harmar Pond highlight the need for further exploration into the ubiquity of trace metal accumulation in roadside environments and the potent impacts of road runoff contamination on near-road ecosystems.

Given the relatively novel combination of methods, it is important to consider the limitations of this approach. Ideally, these results would be replicated in several near-road environments; however, the Pittsburgh region has few lakes and ponds. Harmar Pond was located after extended searching for suitable sedimentary environments and may be the only natural lake this close downwind of Pittsburgh, precluding replication. In addition, sediment metal chemistries (Figure 5) suggest that additional endmembers are required to explain the observed preroad chemistries. This is not an uncommon limitation in mixing analysis, particularly when developing new mixing frameworks. Additional characterization of endmembers in this mixing space can clarify the contributions of contaminants to sediment chemistry. This additional characterization may necessitate a reinterpretation of these results. Perhaps most importantly, these results only apply to specific near-road systems. There will be a temptation to use conditions in near-road environments to excuse long histories of industrial pollution. However, both remain important sources of both legacy and future contamination that degrade environmental systems.

## Supporting Information Available

Sediment core ^210^Pb ages, sedimentation rate, bulk density, concentrations of other metals of interest over time, 1876 map of Harmar Township, and all available details on literature values used for the endmember regions of the mixing space. Sediment core metal concentration data is available here: https://doi.org/10.26022/IEDA/112281 (PDF)
