# Supplementary material for: Pond Sediments Reveal the Increasing Importance of Road Runoff as a Source of Metal Contamination in Industrialized Urban Environments Downwind of Pittsburgh, Pennsylvania (USA)
Source: ACS ES T Water. 2023 Feb 7;3(3):650–8. doi: 10.1021/acsestwater.2c00240 (PMC10034740; doi:10.1021/acsestwater.2c00240)
Supplement: Supplementary file 1 — ew2c00240_si_001.pdf [file ew2c00240_si_001.pdf]

Supporting Information

Pond Sediments Reveal the Increasing Importance of Road Runoff as a Source of Metal Contamination in Industrialized Urban Environments Downwind of Pittsburgh, Pennsylvania (USA)

Authors: Memphis J. Hill*[a], Daniel J. Bain[a], Robert J. Rossi[b], Mark B. Abbott[a]

[a]Department of Geology and Environmental Science, University of Pittsburgh, Pittsburgh, Pennsylvania 15260, United States

[b]PSE Healthy Energy 1440 Broadway, Suite 750, Oakland, California, 94612, United States

*Corresponding author: MEH146@pitt.edu

Pages: 8

Tables: 2

Figures: 4

Depth Interval (cm)	Pb-210 Activity (Bq/g)	Excess Pb-210 Activity (Bq/g)	Age relative to 2016 CE Depth (yr)	Correlated Age 1 sigma Error (yr)	Date at given Depth (CE)	Sedimentation Rate (cm/yr)	Mass Sedimentation Rate (mg cm^{-2} yr^{-1})	Mass Sedimentation Rate Error (%)
0.0				0	2016			
0.25	0.80	0.72	1	2	2015	0.423	85.5	23.6
2.25	0.71	0.66	4	2	2012	0.605	88.25	21.0
4.25	0.68	0.64	8	2	2008	0.541	82.03	21.0
6.25	0.69	0.64	13	3	2003	0.403	70.93	20.9
8.25	0.65	0.60	19	3	1997	0.328	64.20	21.3
12.25	0.41	0.35	24	3	1992	0.724	90.84	23.3
14.25	0.39	0.35	31	3	1985	0.313	76.10	22.1
16.25	0.42	0.36	40	4	1976	0.212	57.63	22.9
18.25	0.33	0.28	48	4	1968	0.254	57.09	24.4
20.25	0.28	0.23	57	5	1959	0.213	52.35	26.9
22.25	0.28	0.22	71	6	1945	0.145	38.09	27.2
24.25	0.24	0.18	91	8	1925	0.098	27.79	31.5
26.25	0.12	0.06	99	9	1917	0.267	53.65	49.6
28.25	0.12	0.07	118	14	1898	0.104	30.95	45.2
30.25	0.10	0.06	169	36	1847	0.039	14.32	88.6

SI Table 1. Harmar Sediment ^{210}Pb Data by Depth and Age calculated using Constant Rate of Supply (CRS) model methods described by Appleby, 1978. 2016 is the year the core was collected.

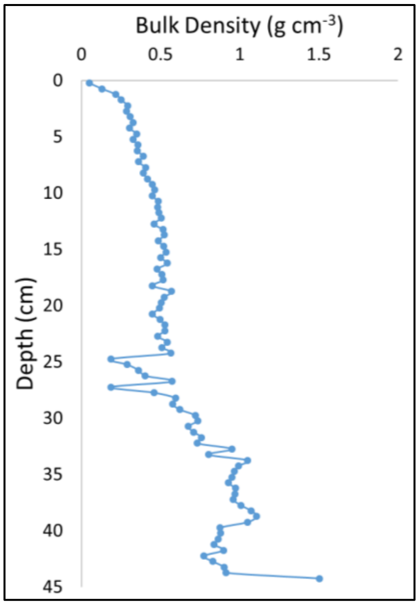

SI Figure 1 Sediment bulk density plotted by depth. Inconsistencies between 25cm and 29cm were caused the plunger slipping during extrusion and do not reflect actual changes in bulk density.

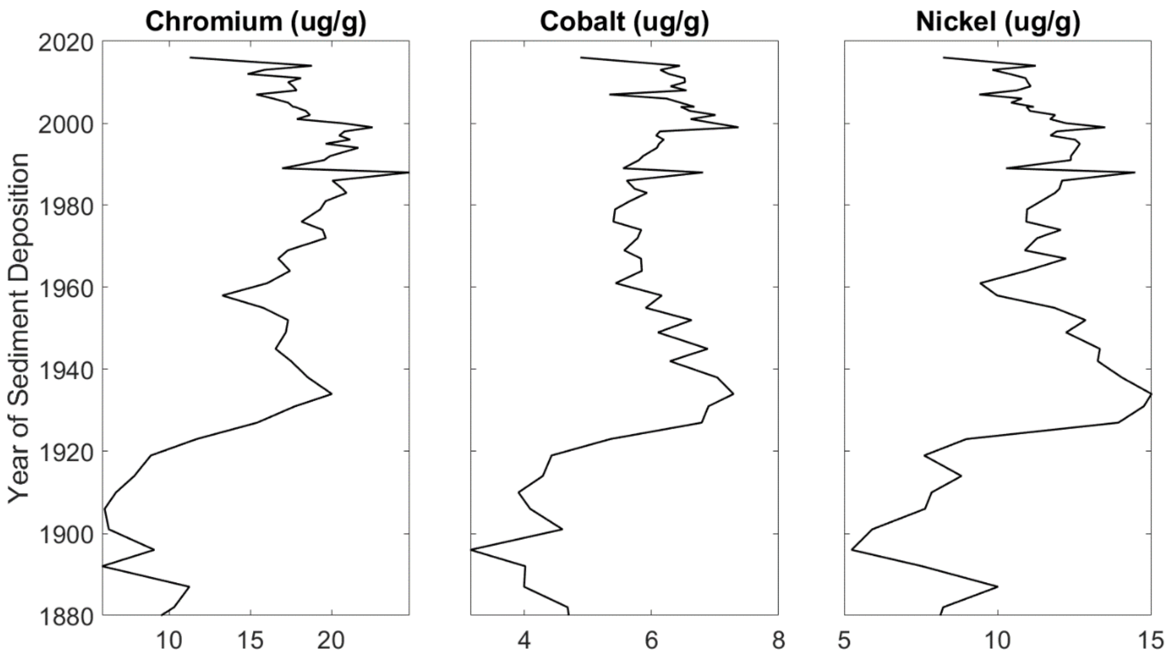

SI Figure 2. Chromium, cobalt, and nickel concentrations in Harmar Pond sediments dated using a ^{210}Pb CRS-based age model.

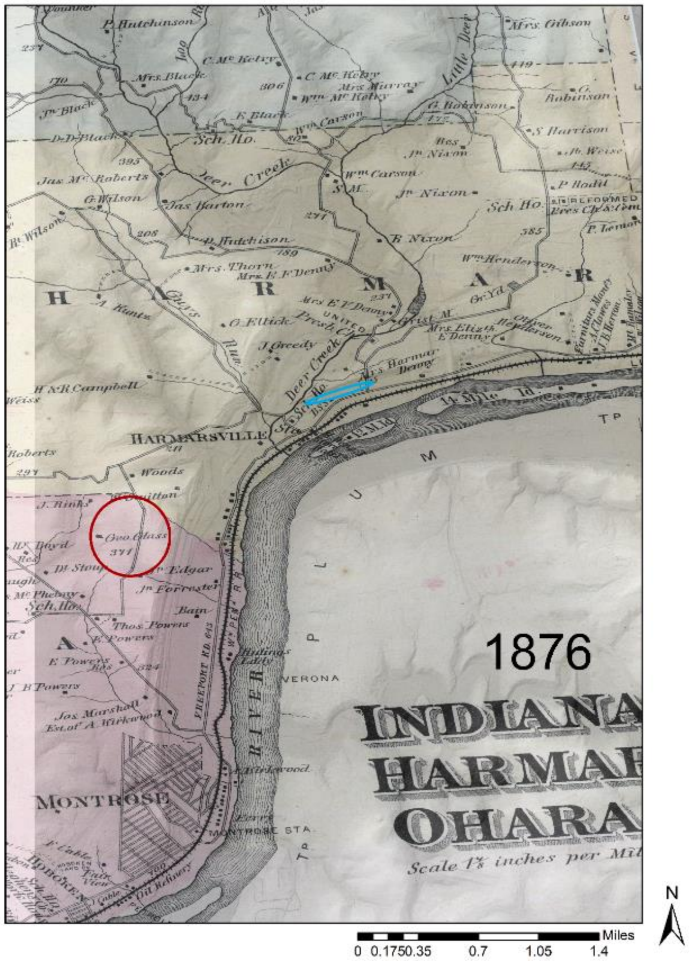

SI Figure 3: Map of Harmar Township in 1876 with Geo Glass circled in red and Harmar Pond outlined in blue. Source: Hopkins, Griffith Morgan, Jr. (1876). *Real estate plat-book of the city of Pittsburgh: from official records, private plans and actual surveys. (1876). Indiana, Harmar, O'Hara. Plate 62*. G. M. Hopkins & Co.: Philadelphia, PA: [digital resource] Historic Pittsburgh. Digital Research Library, University of Pittsburgh., Retrieved from https://digital.library.pitt.edu/islandora/object/pitt:76v01p62. Underlying hillshade derived from: PAMAP Program (2008). PAMAP Program LAS Files (LiDAR Data of Pennsylvania). PA Department of Conservation and Natural Resources. Middletown, PA.

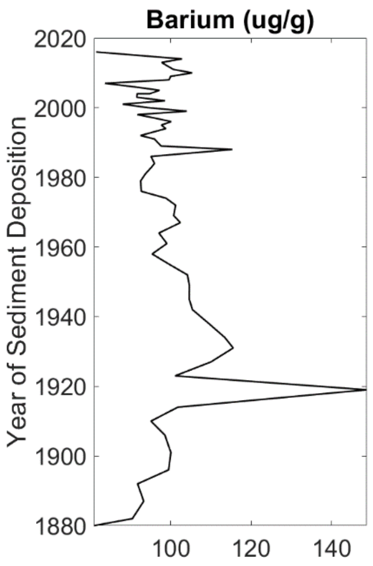

SI Figure 4. Barium concentrations in Harmar Pond sediments dated using a ^{210}Pb CRS-based age model.

First Author	DOI or link	Year published	Sample type	Location	Sample Size	units	Zn	Zn S.D.	Cu	Cu S.D.	Pb	Pb S.D.	Cd	Cd S.D	Cu:Zn	Pb:Cd
R. Breault	10.3133/sir20055184	2003	fine sand	New Bedford, MA, USA	1	ug/g	270.0	N/A	91.0	N/A	420.0	N/A	1.0	N/A	0.337	420.000
R. Breault	10.3133/sir20055184	2003	silt clay	New Bedford, MA, USA	1	ug/g	810.0	N/A	250.0	N/A	1240.0	N/A	3.0	N/A	0.309	413.333
P. Fedotov	10.1016/j.talanta.2014.06.040	2014	road dust	Site 2 Moscow, Russia	4	ug/g	388.0		71.0		75.0		1.0		0.183	75.000
P. Fedotov	10.1016/j.talanta.2014.06.040	2014	road dust	Site 5 Moscow, Russia	4	ug/g	322.0		172.0		132.0		1.0		0.534	132.000
F. Zhang	10.3390/ijerph9051715	2012	roadside topsoil	0m from road, Kathmandu, Nepal	66	mg/kg	69.5	37.85	19.8	7.11	28.8	37.16	0.4	0.37	0.284	75.868
F. Zhang	10.3390/ijerph9051715	2012	roadside topsoil	10m from road,Kathmandu, Nepal	69	mg/kg	81.3	62.1	20.0	6.58	22.9	21.7	0.4	0.44	0.246	65.457
F. Zhang	10.3390/ijerph9051715	2012	roadside topsoil	30m from road, Kathmandu, Nepal	69	mg/kg	81.7	59.23	20.5	7.11	22.2	19.62	0.4	0.4	0.251	61.750
F. Zhang	10.3390/ijerph9051715	2012	roadside topsoil	50m from road, Kathmandu, Nepal	72	mg/kg	71.4	46.55	19.3	7.59	17.7	14.49	0.4	0.7	0.271	49.083
F. Zhang	10.3390/ijerph9051715	2012	roadside topsoil	100m from road, Kathmandu, Nepal	66	mg/kg	77.5	53.35	20.4	6.14	21.7	20.04	0.3	0.28	0.263	65.667
B. Wei	10.1016/j.microc.2009.09.014	2010	road dust	Hangzhou, China	25	mg/kg	321.4		116.0		202.2		1.6		0.361	127.145
B. Wei	10.1016/j.microc.2009.09.014	2010	road dust	Shanghai, China	273	mg/kg	733.8		196.8		294.9		1.2		0.268	239.756
B. Wei	10.1016/j.microc.2009.09.014	2010	road dust	Guangzhou, China	30	mg/kg	586.0		176.0		240.0		2.4		0.300	99.585
B. Wei	10.1016/j.microc.2009.09.014	2010	road dust	Urumqi, China	169	mg/kg	294.5		94.5		53.5		1.2		0.321	45.752
Y. Nazzal	10.1007/s10661-012-2672-3	2013	road dust	Greater Toronto Area, ON, Canada	42	ppm	232.8		162.2		182.8		0.5		0.697	357.031
M. Dietrich	https://doi.org/10.1016/j.scitotenv.2017.09.246	2018	road dust	Middletown, Ohio	14	ppm	374.5	237.57	28.23	10.66	85.0	63.89	0.21	0.14	0.075	404.571
J. Steuer	pubs.usgs.gov/wri/1997/4242/report.pdf	1997	basin outlet, total recoverable	Marquette, MI, USA	14	ug/L	111.0		22.0		49.0		0.6		0.198	81.667
R. Bannerman	https://doi.org/10.2166/wst.1993.0426	1993	storm-sewer outfall	Monroe residential area, Madison, WI, USA	7	ug/L	203.0		16.0		32.0		0.4		0.079	80.000
R. Bannerman		1993	storm-sewer outfall	Syene industrial area, Madison, WI, USA	7	ug/L	265.0		28.0		25.0		1.0		0.106	25.000
R. Bannerman		1993	parking lot runoff	Monroe commerical area, Madison, WI, USA	5	ug/L	178.0		15.0		22.0		0.6		0.084	36.667
M. Kayhanian	10.1016/j.jenvman.2006.09.024	2007	runoff mean	California highways, CA, USA	635	ug/L	187.1	199.8	33.5	31.6	47.8	151.3	0.7	1.6	0.179	68.286
	10.1016/j.jenvman.2006.09.024	2007	runoff median	California highways, CA, USA	635	ug/L	111.2		21.2		12.7		0.4		0.191	28.864
L. Tiefenthaler	https://ftp.sccwrp.org/pub/download/DOCUMENTS/T	2001	simulated rainfall wash-off pre-cleaning	Long Beach, CA, USA	42	ug/L	530.0	169.7	37.5	13.4	20.0	11.3	0.7	0.9	0.071	28.571
L. Tiefenthaler	echnicalReports/343_characteristics_of_parkinglot_ru	2001	simulated rainfall wash-off post-cleaning	Long Beach, CA, USA	42	ug/L	220.0	85.1	32.0	9.2	35.0	9.2	1.3	1.4	0.145	26.923
L. Tiefenthaler	noff.pdf	2001	simulated rainfall wash-off 2 months post-cleaning	Long Beach, CA, USA	42	ug/L	620.0	60.4	40.3	7.2	41.8	10.6	2.5	1.4	0.065	16.720
W. Selbig	https://pubs.usgs.gov/sir/2007/5156/pdf/SIR_2007-	2007	Stormwater from control basin during calibration	Madison, WI	34,34,32,18*	ppb	65.9	107.6	13.4	7.06	7.9	5.34	3.9	14.03	0.203	2.044
W. Selbig	5156.pdf	2007	Stormwater from control during treatment	Madison, WI	35,35,34,19*	ppb	68.7	43.47	20.0	15.83	11.8	9.1	2.1	7.73	0.292	5.692
R. Finkelman	https://www.osti.gov/servlets/purl/891305	2006	Pittsburgh coal	Washington County, PA, USA	1	ppm	22.4	N/A	6.2	N/A	4.7	N/A	0.1	N/A	0.275	42.936
		2006	Pittsburgh coal	Washington County, PA, USA	1	ppm	47.9	N/A	29.6	N/A	13.6	N/A	0.2	N/A	0.618	70.103
		2006	Pittsburgh coal	Washington County, PA, USA	1	ppm	59.1	N/A	25.9	N/A	14.0	N/A	0.2	N/A	0.438	69.652
		2006	Pittsburgh coal	Washington County, PA, USA	1	ppm	18.6	N/A	8.4	N/A	5.0	N/A	0.1	N/A	0.452	43.596
		2006	Pittsburgh coal	Washington County, PA, USA	1	ppm	98.7	N/A	59.7	N/A	36.1	N/A	0.4	N/A	0.605	84.941
		2006	Pittsburgh coal	Washington County, PA, USA	1	ppm	21.7	N/A	17.0	N/A	6.1	N/A	0.1	N/A	0.783	64.693
R. Finkelman	https://www.osti.gov/servlets/purl/891306	2006	Upper Freeport coal	Grant Count, WV, USA	1	ppm	37.2	N/A	15.4	N/A	10.2	N/A	0.1	N/A	0.414	93.578
R. Finkelman	https://www.osti.gov/servlets/purl/891307	2006	Upper Freeport coal	Preston County, WV, USA	1	ppm	17.6	N/A	12.7	N/A	9.2	N/A	0.1	N/A	0.722	119.380
R. Finkelman	https://www.osti.gov/servlets/purl/891308	2006	Upper Freeport coal	Preston County, WV, USA	1	ppm	22.2	N/A	4.8	N/A	6.4	N/A	0.1	N/A	0.214	80.405
R. Finkelman	https://www.osti.gov/servlets/purl/891309	2006	Upper Freeport coal	Preston County, WV, USA	1	ppm	15.3	N/A	10.1	N/A	7.6	N/A	0.1	N/A	0.660	132.578
M. Dourson	https://doi.org/10.1080/10962247.2016.1180328	2016	Petcoke	Chicago, IL, USA	6	ppm	9.0	15.0	2.0	1.3	2.1	4.4	0.2	0.011	0.222	9.130
M. Dourson		2016	Petcoke	Chicago, IL, USA	12	ppm	7.0	7.9	1.9	3.0	3.0	4.3	0.1	0.0065	0.271	27.273
E. Weitkamp	://doi.org/10.1016/j.atmosenv.2005.06.028	2005	PM 2.5 coke emissions	Pittsburgh, PA, USA	7	% mass	0.1	0.0	0.0	0.0	0.1	0.0	0.0	0.0005	0.192	22.564
M. Bettinelli	10.1006/mchj.1998.1600	1998	Coal Fly Ash	Italy	3	ug/g	233.0	3.0	117.0	3.0	78.7	2.8	1.0	0.03	0.502	77.921
J. Burgess-Conforti	https://scholarworks.uark.edu/etd/1842/	2016	DFGD by-product applied to soil	Fayetteville, AR, USA	3	ug/kg	10800.0		5500.0		23.5		0.1		0.509	235.000
J. Burgess-Conforti		2016	dry flue gas desulfurization (DFGD) by-product		1	mg/kg	140.8	N/A	73.2	N/A	0.2	N/A	0.4	N/A	0.519	0.455
J. Burgess-Conforti		2016	Class-C fly ash from Flint Creek power plant	Benton County, AR, USA	1	mg/kg	50.5	N/A	49.6	N/A	108.2	N/A	0.7	N/A	0.982	152.451
R. Finkelman	https://www.osti.gov/servlets/purl/891308	2006	Coal Fly Ash	Pulaski County, KY	1	ppm	81.1	N/A	111.0	N/A	28.1	N/A	0.3	N/A	1.369	104.074
R. Finkelman	https://www.osti.gov/servlets/purl/891315	2006	Coal Fly Ash	Pulaski County, KY	1	ppm	220.3	N/A	236.5	N/A	160.9	N/A	1.8	N/A	1.073	89.500
R. Finkelman	https://www.osti.gov/servlets/purl/891316	2006	Coal Fly Ash	Pulaski County, KY	1	ppm	222.1	N/A	225.7	N/A	165.3	N/A	1.8	N/A	1.016	93.000
R. Finkelman	https://www.osti.gov/servlets/purl/891317	2006	Coal Fly Ash	Pulaski County, KY	1	ppm	290.6	N/A	262.7	N/A	187.1	N/A	2.3	N/A	0.904	80.000
R. Finkelman	https://www.osti.gov/servlets/purl/891318	2006	Coal Fly Ash	Pulaski County, KY	1	ppm	251.8	N/A	242.9	N/A	178.8	N/A	13.8	N/A	0.965	12.968
R. Finkelman	https://www.osti.gov/servlets/purl/891319	2006	Coal Fly Ash	Pulaski County, KY	1	ppm	512.8	N/A	363.1	N/A	245.0	N/A	3.9	N/A	0.708	63.636
R. Finkelman	https://www.osti.gov/servlets/purl/891320	2006	Coal Fly Ash	Pulaski County, KY	1	ppm	321.1	N/A	278.8	N/A	194.2	N/A	2.4	N/A	0.868	80.357
R. Finkelman	https://www.osti.gov/servlets/purl/891322	2006	Coal Fly Ash	Pulaski County, KY	1	ppm	287.7	N/A	267.9	N/A	184.3	N/A	2.2	N/A	0.931	82.000
H. Mielke	https://ehp.niehs.nih.gov/doi/epdf/10.1289/ehp.01109 973	2001	Exterior House Paint, median	New Orleans, LA, USA	31	ug/g	31101.0		21.0		35248.0		27.0		0.001	1305.481
D. Bleiwas	https://pubs.usgs.gov/of/2010/1131/pdf/OF10-1131.pdf	2010	1999 combined air stack and fugitive emissions	Monaca, PA	not published	kg/yr	190700		14		4100		200		0.000073	20.5
			2003 combined air stack and fugitive emissions	Monaca, PA	not published	kg/yr	202400		11		4000		300		0.000054	13.33333
			2005 combined air stack and fugitive emissions	Monaca, PA	not published	kg/yr	200700		15		3800		200		0.000075	19
			flue dust recovered from calcining of concentrate	Monaca, PA	not published	%	54		0.4		1.4		0.41		0.007407	3.414634
			Zinc Retort furnaces	Donora zinc plant	not published	lbs/day	24000				100		220			0.454545
EPA TRI Facility Report	https://enviro.epa.gov/facts/tri/ef-facilities/#/Chemical/15061ZNCCR300FR		1998 combined air stack and fugitive emissions	Monaca, PA	not published	lbs/yr	425460		30		10530		633		0.000071	16.63507
			2004 combined air stack and fugitive emissions	Monaca, PA	not published	lbs/yr	441818		25		8502		550		0.000057	15.45818
			2006 combined air stack and fugitive emissions	Monaca, PA	not published	lbs/yr	438568		25		8375		535		0.000057	15.65421
			2007 combined air stack and fugitive emissions	Monaca, PA	not published	lbs/yr	580802		24		11316		553		0.000041	20.46293
			2008 combined air stack and fugitive emissions	Monaca, PA	not published	lbs/yr	573352		15		10839		265		0.000026	40.90189
			2009 combined air stack and fugitive emissions	Monaca, PA	not published	lbs/yr	460098		13		8110		245		0.000028	33.10204
			2010 combined air stack and fugitive emissions	Monaca, PA	not published	lbs/yr	411730		35		8826		257		0.000085	34.34241
M. Ketterer	https://doi.org/10.1016/S0883-2927(00)00029-9	2001	Atmospheric particulate matter collected on glass fiber filters at locations X08 (central Palmerton) and AQ32 (2 km east of the EAF processing plant)	Palmerton, PA	10	ppm	51000		2640		7490		420		0.051765	17.83333
H. Schrenk	https://collections.nlm.nih.gov/catalog/nlm:nlmuid-31320170R-bk	1949	ore roasting	Donora, PA	not published	mg/m^3 of air	4.8				0.11		0.05			2.2
					not published		19.4				0.32		0.19			1.684211
			No. 5 Cottrell exhaust stack (sinter plant)	Donora, PA	not published	mg/m^3 of air	62.2				26		32.8			0.792683
					not published		26.9				23.6		20			1.18
					not published		26.3				71.6		19.4			3.690722
					not published		64				44.1		22.4			1.96875
					not published		45.7				70.7		15.3			4.620915
			East end of No. 4 spelter, collected inside of hood table 40	Donora, PA	not published	mg/m^3 of air	9.2				0.07		0.22			0.318182
					not published		27.5				0.09		0.25			0.36
					not published		35.5				0.19		0.21			0.904762
					not published		15.7				0.17		0.26			0.653846
					not published		96				0.73		1.66			0.439759
					not published		124				0.51		2.31			0.220779
					not published		120.5				0.39		0.9			0.433333
					not published		83				0.5		0.5			1
					not published		96.7				0.65		3.13			0.207668
					not published		92				0.24		1.34			0.179104
					not published		23				0.14		0.39			0.358974
					not published		57				0.15		0.42			0.357143
					not published		29.9				0.27		0.09			3
					not published		109				0.93		0.34			2.735294
					not published		73.5				0.95		0.26			3.653846
					not published		48				0.18		0.7			0.257143
					not published		45.4				0.33		0.33			1

*SI Table 2. Literature values for end-member metal ratios. Standard deviations are listed when available. Copper values not listed in 2010 USGS report were found in TRI reports from the matching years. *Values from Selbig, 2007 have different sample sizes for each metal, n is listed in this order; Zn, Cu, Pb, Cd.*
